# Localized waves in elastic plates with perturbed honeycomb arrays of constraints

**DOI:** 10.1098/rsta.2021.0404

**Published:** 2022-09-05

**Authors:** S. G. Haslinger, S. Frecentese, G. Carta

**Affiliations:** ^1^ Department of Mathematical Sciences, University of Liverpool, Liverpool L69 7ZL, UK; ^2^ University of Cagliari, Department of Mechanical, Chemical and Materials Engineering, Cagliari 09123, Italy

**Keywords:** elastic plates, localized waves, honeycomb arrays, perturbations, valley Chern number

## Abstract

In this paper, we study wave propagation in elastic plates incorporating honeycomb arrays of rigid pins. In particular, we demonstrate that topologically non-trivial band-gaps are obtained by perturbing the honeycomb arrays of pins such that the ratio between the lattice spacing and the distance of pins is less than 3; conversely, a larger ratio would lead to the appearance of trivial stop-bands. For this purpose, we investigate band inversion of modes and calculate the valley Chern numbers associated with the dispersion surfaces near the band opening, since the present problem has analogies with the quantum valley Hall effect. In addition, we determine localized eigenmodes in strips, repeating periodically in one direction, that are subdivided into a topological and a trivial section. Finally, the outcomes of the dispersion analysis are corroborated by numerical simulations, where a time-harmonic point source is applied to a plate with finite arrays of rigid pins to create localized waves immune to backscattering.

This article is part of the theme issue ‘Wave generation and transmission in multi-scale complex media and structured metamaterials (part 1)’.

## 1. Introduction

Topologically protected wave propagation in elastic metamaterials has attracted increasing attention in the scientific community in recent years, due to the possibility of creating waveguides that are immune to backscattering in the presence of defects, such as impurities or sharp corners. The idea was first proposed in quantum mechanics [1,2] and later extended to photonic [2–6] and acoustic [7–12] media.

In elasticity, different classes of passive models have been proposed to realize topologically protected edge states, such as lattices [13–15] and plates [16,17]. In the latter type of structure, topological supernetworks with tunable directionality, based on combinations of multi-directional energy splitters, have been designed in [18–20] using group theory, topological concepts and tunnelling phenomena. Edge waves have been observed in discrete lattices including tilted resonators in [21,22], while wave localization in lattices of Rayleigh beams has been investigated in [23–25]. Localized folding motions at the boundaries of origami and kirigami structures have been connected to topological polarization in [26]. Topological properties of rotational waves in granular crystals have been discussed in [27]. Valley anisotropy has been observed in [28], where a chiral system consisting of hard spiral scatterers embedded in a soft material matrix has been studied.

The systems mentioned above, comprising only passive elements, do not break time-reversal symmetry. The latter can be broken if active components are also incorporated or if an external field with a momentum bias is applied to the system. For instance, in [29], a lattice model has been employed to describe topologically protected edge modes in microtubules, present in eukaryotic cells, where time-reversal symmetry can be broken by weak magnetic properties of the tubulin proteins. Gyroscopic action can also be used in this framework to create topological insulators, as demonstrated in [30–37] for elastic lattices and in [38,39] for elastic plates.

In this paper, we consider an elastic plate constrained by a periodic arrangement of honeycomb arrays of rigid pins. Dirac cones are broken by perturbing the positions of the pins, and as a consequence a stop-band is generated. Following the concept proposed in [40] and developed for a honeycomb array of dielectric cylinders in a material with different dielectric properties, we alter the locations of the pins to create either a topological or a trivial band-gap in proximity to the broken Dirac cone.

The topology of Dirac cones in pinned elastic plates has been extensively investigated in [41,42], where unidirectional trapped modes have also been observed. Wave transmission in elastic plates incorporating periodic arrays of rigid pins or masses has been studied in [43–45], while localization phenomena produced by semi-infinite grating stacks of pins or different types of oscillators have been examined in [46–48]. The possibility of attaching active sources to a plate with the purpose of designing an optimal cloaking device has been proposed in [49]. The effect of cavities on the band diagram of a platonic crystal has been analysed in [50,51], both numerically and experimentally, while the band structures of thin and thick plates connected to periodic systems of spring-mass resonators have been obtained in [52,53]. Homogenized models to describe flexural vibrations localized in single or double rings of spring-mass resonators have been formulated in [54] and [55], respectively, based on pointwise descriptions of dynamic fields of the meso-scale type [56,57].

The paper is organized as follows. After presenting the model in §2, we discuss the dispersion properties for both the unperturbed and perturbed systems in §3, focusing the attention on the band inversion of modes. Then, in §4, we calculate the valley Chern numbers to distinguish between topological and trivial regimes. In §5, we show localized interfacial modes in periodic strips divided into a topological and a trivial region, and in §6, we illustrate topologically robust waves that are immune to backscattering. Finally, in §7, we provide concluding remarks.

## 2. Motivation for the work and description of the model

**Figure 1.**
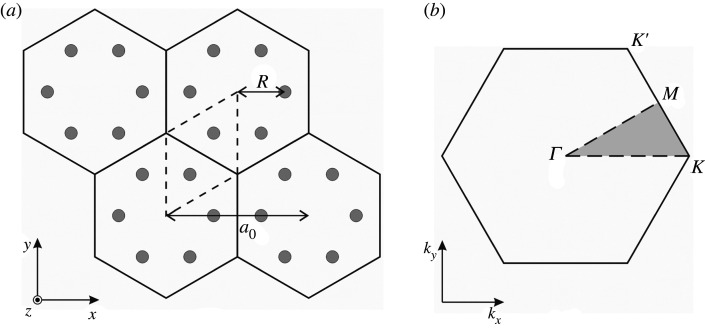
(*a*) Unperturbed honeycomb array, that can be generated by either a rhombic (dashed) or hexagonal macrocell. In the latter case, it is characterized by the lattice parameter $a_{0}/R=3$, where $a_{0}$ is the spacing between the centres of neighbouring hexagonal macrocells, and R is the distance of an individual cylinder from its cell’s centre. (*b*) First Brillouin zone for the triangular lattice in the reciprocal space. Dirac cones arise at the K and $K^{\prime }$ points.

We consider the Kirchhoff–Love elastic plate analogue of a topological photonic crystal, made purely of conventional dielectric material, which was proposed in [40]. In particular, in [40], a honeycomb array of cylinders with dielectric constant $\varepsilon_{d}$ is embedded within a surrounding medium characterized by different dielectric constant $\varepsilon_{a}$. The unperturbed honeycomb lattice (generated by a primitive rhombic unit cell) is illustrated in figure 1*a*, where neighbouring cylinders are separated by R. Introducing the lattice constant $a_{0}$, the system is equivalent to a triangular lattice of hexagonal cells composed of six neighbouring cylinders when $a_{0}/R=3$. Taking this larger hexagonal macrocell as the unit cell, the Dirac cones arising at the K and $K^{\prime }$ points in the first Brillouin zone of the honeycomb lattice, shown in figure 1*b*, are folded into doubly degenerate Dirac cones at the Γ point ($k_{x}=k_{y}=0$) for the macrocell treatment [41,58]. By varying the lattice parameter $a_{0}/R$, the triangular lattice of hexagonal cells is deformed in such a way as to preserve both the triangular lattice and the $C_{6}$ symmetry, but leads to anisotropy, including the opening of a non-trivial band-gap at the quadruply degenerate Dirac point.

Here, we study the same periodic structure but replace the dielectric photonic crystal with a platonic crystal, where a thin elastic plate is structured with an array of rigid pins (zero displacement at point scatterers). Using the biharmonic model for flexural wave propagation in Kirchhoff–Love plates, the governing equation for the out-of-plane displacement u(r) is expressed by

2.1 $$\Delta^{2}u(r)-\frac{\rho h\omega^{2}}{D}u(r)=0,$$

where Δ represents the Laplacian operator, ρ is the density (mass per unit volume), h is the thickness and $D=Eh^{3}/(12(1-\nu^{2}))$ is the flexural rigidity of the plate, with E and ν being the Young’s modulus and Poisson’s ratio, respectively. In addition, r is the position vector and ω is the angular frequency. The spectral parameter β is often employed, and its relations with both ω and the frequency f are given by

2.2 $$\beta^{2}=\omega \sqrt{\frac{\rho h}{D}},\;f=\frac{\omega }{2\pi }.$$

We point out that the spectral parameter β has the dimensions of a wavenumber.

**Figure 2.**
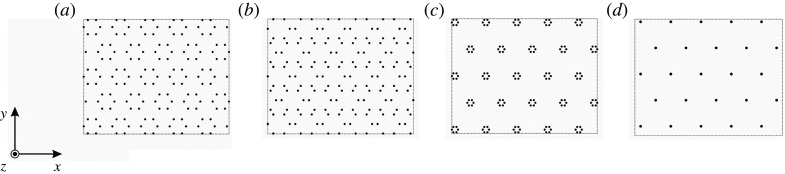
Comparison of perturbed honeycomb lattices: (*a*) $a_{0}/R=3.5$, (*b*) $a_{0}/R=2.5$, (*c*) $a_{0}/R=10$ and (*d*) $a_{0}/R=1$.

Regarding the honeycomb array of point constraints, by increasing $a_{0}/R$ we squeeze the rigid pins closer together (see figure 2*a* for $a_{0}/R=3.5$), producing a triangular lattice of regular hexagonal cells. Reducing the lattice parameter has the opposite effect, stretching the triangular lattice’s constituent hexagonal cells (see figure 2*b* for the case $a_{0}/R=2.5$). Note that the perturbation of figure 1*a*’s pure honeycomb structure results in irregular hexagons between the triangular lattice’s regular hexagonal macrocells, whose size and nature depend on the choice of whether to reduce or increase $a_{0}/R$. The limiting cases are shown in figures 2*c*,*d*: in part (*c*), we illustrate the case $a_{0}/R=10$, which demonstrates that in the limit as $a_{0}/R\to \infty$, the lattice approaches the pure hexagonal Bravais lattice; the same result occurs for the limiting case $a_{0}/R\to 1$, shown in part (*d*).

## 3. Dispersion properties

The dispersion diagrams and eigenmodes of the unperturbed and perturbed structured plates are determined numerically using the finite-element package *Comsol Multiphysics*. To this aim, Bloch–Floquet conditions are applied at the boundaries of the periodic cell [38,39,46]. In the following, we consider an aluminium plate having Young’s modulus E=70 GPa, Poisson’s ratio ν=0.33, density $\rho =2700\;{\mathrm{kg}\;m}^{-3}$ and thickness h=0.002 m. The lattice constant is taken as $a_{0}=1\;m$.

### (a) Unperturbed array of rigid pins

**Figure 3.**
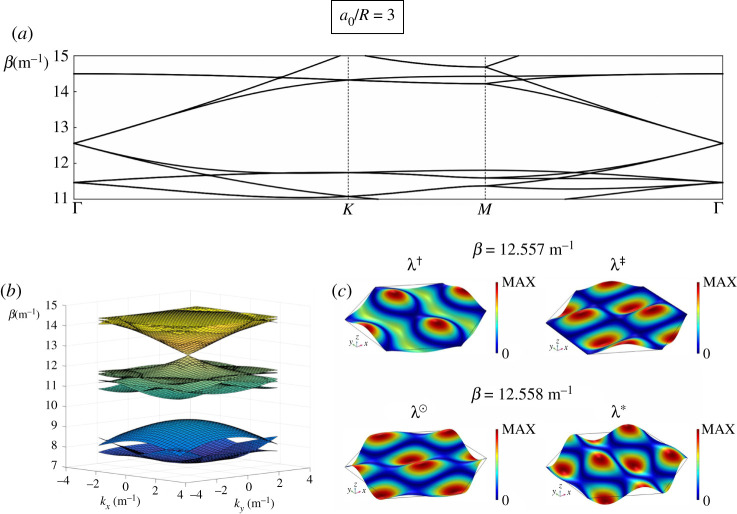
(*a*) Dispersion diagram for the unperturbed honeycomb lattice (generated equivalently by rhombic or hexagonal macrocell) with $a_{0}/R=3$, evaluated along the path ΓKMΓ and shown in the range $\beta \in [11,15]\;({\text{m}}^{-1})$. (*b*) Dispersion surfaces for the same structure, shown in the range $\beta \in [7,15]\;({\text{m}}^{-1})$. (*c*) Eigenmodes associated with the Dirac point at $\beta ≃12.56\;{\text{m}}^{-1}$. For $\beta =12.557\;{\text{m}}^{-1}$ (slightly below the Dirac point), the two modes are denoted by $\lambda^{†}$ and $\lambda^{\text{‡}}$; for $\beta =12.558\;{\text{m}}^{-1}$ (slightly above the Dirac point), we use $\lambda^{⊙}$ and $\lambda^{*}$. (Online version in colour.)

The dispersion diagram for the unperturbed honeycomb system with the hexagonal macrocell defined by $a_{0}/R=3$, computed along the path ΓKMΓ (see figure 1*b*), is shown in figure 3*a*. The corresponding dispersion surfaces near Γ are illustrated in figure 3*b*, where we plot the spectral parameter β on the vertical axis versus the wavevector components $k_{x}$ and $k_{y}$ on the horizontal axes. Note that the presence of the rigid pins leads to a low-frequency band-gap for $\beta <7.25\;{\text{m}}^{-1}$. We also observe another significant band-gap for this system for $9.34\;{\text{m}}^{-1}\leq \beta \leq 10.88\;{\text{m}}^{-1}$. The most striking feature of figure 3*a*,*b* is the quadruply degenerate Dirac point at $\Gamma =(k_{x},k_{y})=(0,0)$ for $\beta ≃12.56\;{\text{m}}^{-1}$, where two doubly degenerate Dirac cones meet (the dispersion diagram in part (*a*) is illustrated in the interval β∈[11,15] (${\text{m}}^{-1}$) to better illustrate the Dirac point). This is analogous to the double Dirac cones identified in [40] for the photonic honeycomb crystal, and is also reminiscent of the multiply degenerate Dirac points observed in [41] for elastic plates pinned at points of a hexagonal lattice (although in [41] an additional flat band surface, passing through the Dirac-like point, is also present).

In figure 3*c*, we illustrate the system’s Bloch modes in proximity of the Dirac point frequency at the Γ-point. The two modes for $\beta =12.557\;{\text{m}}^{-1}$ (or, equivalently, f=78.156 Hz), which are associated with the apex of the lower cone in figure 3*b*, are denoted by $\lambda^{†}$ and $\lambda^{\text{‡}}$. By contrast, the two modes for $\beta =12.558\;{\text{m}}^{-1}$ (corresponding to f=78.159 Hz), which belong to the upper cone in figure 3*b*, are indicated by $\lambda^{⊙}$ and $\lambda^{*}$.

### (b) Perturbed arrays of rigid pins

For the analogous photonic crystal discussed in [40], reducing the lattice parameter (namely, taking $a_{0}/R<3$) opens a global band-gap near the Dirac point, and a band inversion takes place—this is referred to as the *topological* regime in [40]. Conversely, for the opposite type of perturbation (such that $a_{0}/R>3$), no band inversion takes place, and this is called the *trivial* regime. These contrasting scenarios are determined by the $p_{\pm }$ and $d_{\pm }$ states for the electromagnetic dielectric case. Specifically, solving the Maxwell equations yields harmonic transverse magnetic modes that are supported by the hexagonal cells, which act as ‘artificial atoms’ in the present geometry. The modes exhibit orbital-like p- and d-wave shapes, hence the terminology adopted in [40] to distinguish between the types of mode, and form photonic bands.

**Figure 4.**
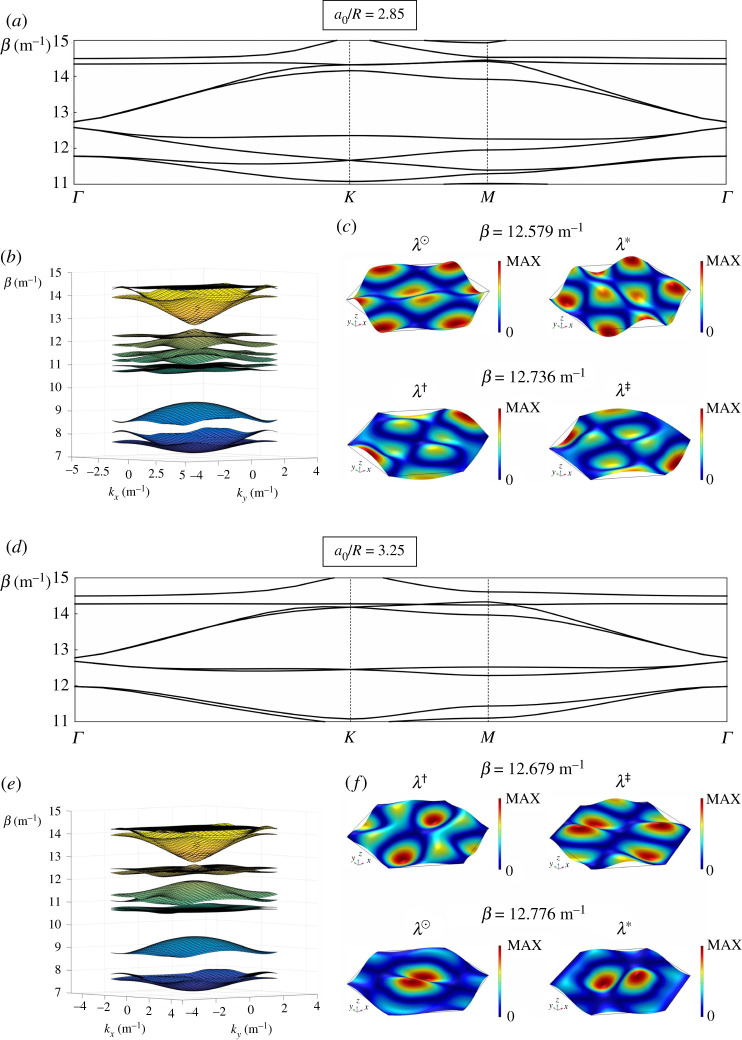
Results for perturbed honeycomb lattices, produced by varying the lattice parameter: (*a*–*c*) $a_{0}/R=2.85$, (*d*–*f*) $a_{0}/R=3.25$. (*a*,*d*) Dispersion diagrams. (*b*,*e*) Dispersion surfaces. (*c*,*f*) Eigenmodes in the vicinity of the band opening around the Dirac point at Γ=(0,0); the modes similar to those of the unperturbed honeycomb array in figure 3*c* are labelled in the same way with $\lambda^{†}$, $\lambda^{\text{‡}}$, $\lambda^{⊙}$ and $\lambda^{*}$. (Online version in colour.)

For the platonic case here, perturbing the array of pins leads to the opening of a band-gap around the Dirac point at Γ=(0,0). When $a_{0}/R=2.85$ (figure 4*a*,*b*), the stop-band is in the range $\beta \in [12.58,12.74]\;({\text{m}}^{-1})$. On the other hand, when $a_{0}/R=3.25$ (figure 4*d*,*e*), the band-gap is narrower: $\beta \in [12.68,12.78]\;({\text{m}}^{-1})$.

The eigenmodes demonstrate something analogous to the photonic crystal’s band inversion. For $a_{0}/R=3.25$, equivalent to the *trivial* regime in [40], the modes below ($\lambda^{†}$ and $\lambda^{\text{‡}}$) and above ($\lambda^{⊙}$ and $\lambda^{*}$) the band opening at Γ=(0,0) are similar to those found for the unperturbed array of pins (compare figures 3*c* and 4*f*). Conversely, for the *topological* regime ($a_{0}/R=2.85$), the modes $\lambda^{⊙}$ and $\lambda^{*}$ arise at a lower frequency than $\lambda^{†}$ and $\lambda^{\text{‡}}$, as shown in figure 4*c*. A band inversion takes place around the Dirac point, and so a non-trivial band-gap is obtained, by perturbing the periodicity with $a_{0}/R<3$.

## 4. Calculation of valley Chern number

In order to compute the valley Chern number, we follow a procedure similar to that developed in [28,30]. The considered elastic periodic plate, constrained by arrays of rigid pins, is a continuous structure, that is discretized with the finite-element method to determine numerically the dispersion diagrams. For each dispersion surface, we can obtain the eigenvector W=W(k) corresponding to any value of the wavevector $k={\left(k_{x},k_{y}\right)}^{T}$ and to the specific value of the frequency ω=ω(k) associated with that dispersion surface.

Since the system has time-reversal symmetry, the integration of the Berry curvature Ω(k) (defined below) over the Brillouin zone is zero; however, Ω(k) is localized near the valleys (in particular, at the K and $K^{\prime }$ points), hence the integration over a small area around the valley results in a non-zero value [28]. This integration is denoted as the *valley Chern number* [28]:

4.1 $$C_{v}=\frac{1}{2\pi }\int_{S}\Omega (k)d^{2}k,$$

where S is a small area around the valley.

**Figure 5.**
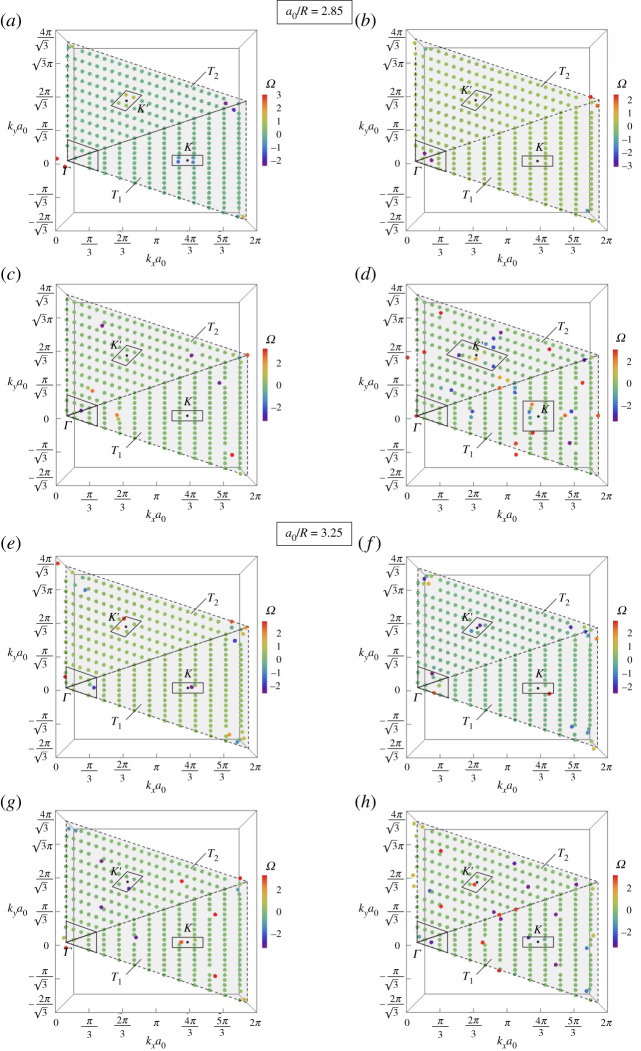
Maps of the Berry curvature Ω(k) for the lattice parameters (*a*–*d*) $a_{0}/R=2.85$ and (*e*–*h*) $a_{0}/R=3.25$, corresponding to the (*a*,*e*) 8th, (*b*,*f*) 9th, (*c*,*g*) 10th and (*d*,*h*) 11th dispersion surface. The triangles $T_{1}$ and $T_{2}$ are the areas of pertinence of the K and $K^{\prime }$ points, respectively. The areas around the points Γ, K and $K^{\prime }$, delimited by solid thick lines, indicate the regions where the valley Chern numbers have been calculated. (Online version in colour.)

The valley Chern number is calculated as follows. First, we consider the regions pertinent to the K and $K^{\prime }$ points in the reciprocal space, corresponding to the triangles indicated by $T_{1}$ and $T_{2}$ in figure 5. More specifically, the triangle $T_{1}$ has its vertices at the points $\left(k_{x},k_{y})=(0,0\right)$, $\left(2\pi /a_{0},-2\pi /(\sqrt{3}a_{0})\right)$ and $\left(2\pi /a_{0},2\pi /(\sqrt{3}a_{0})\right)$, while for the triangle $T_{2}$ the vertices are at the points $\left(k_{x},k_{y})=(0,0\right)$, $\left(2\pi /a_{0},2\pi /(\sqrt{3}a_{0})\right)$ and $\left(0,4\pi /(\sqrt{3}a_{0})\right)$. Then, we discretize each triangle into triangular and square facets, whose base and height are sufficiently small (in our calculations, they are equal to $[2\pi /a_{0}]/12$ and $[2\pi /(\sqrt{3}a_{0})]/12$, respectively). For each square facet (for the triangular ones similar considerations apply, taking into account three points instead of four), we determine the eigenvectors at the k-points corresponding to the vertices of the square facet, indicated by $P_{1}$, $P_{2}$, $P_{3}$ and $P_{4}$, taken along a counter-clockwise path. The Berry curvature is evaluated as [30]

4.2 Ω(k)=−Im(log⁡[⟨W(P1)|W(P2)⟩⟨W(P2)|W(P3)⟩⟨W(P3)|W(P4)⟩⟨W(P4)|W(P1)⟩⟨W(P1)|W(P1)⟩⟨W(P2)|W(P2)⟩⟨W(P3)|W(P3)⟩⟨W(P4)|W(P4)⟩]).

In the formula above,

4.3 $$\langle W(P_{i})|W(P_{j})\rangle =\int_{A}W^{*}(k{|}_{P_{i}})\cdot W(k{|}_{P_{j}})dA,$$

where ${\;}^{*}$ denotes the complex conjugate and A is the area of the periodic cell. Note that the Berry curvature in (4.2) is intended to be associated with the eigenvector k at the middle point of the square facet. For the triangular facets, the Berry curvature is similarly evaluated at their centre.

In figure 5, we show the colour maps of the Berry curvature over the regions $T_{1}$ and $T_{2}$, relative to the honeycomb arrays with lattice parameters $a_{0}/R=2.85$ (parts (*a*)–(*d*)) and $a_{0}/R=3.25$ (parts (*e*)–(*h*)). In particular, four dispersion surfaces from figure 4*b*,*e* have been considered: the 8th (figure 5 parts (*a*) and (*e*)), the 9th (parts (*b*) and (*f*)), the 10th (parts (*c*) and (*g*)) and the 11th (parts (*d*) and (*h*)). For both values of the lattice parameter, the eighth and ninth dispersion surfaces lie below the band-gap generated by the perturbation imposed on the pins’ locations, and they converge to each other at point Γ. Conversely, the 10th and 11th dispersion surfaces are above the same band-gap and share a common point at Γ. Looking at figure 5, we observe clear localizations of Ω(k) at particular positions of the reciprocal space, in particular at Γ, K and $K^{\prime }$.

The values of the valley Chern number $C_{v}$ for the lattice parameter $a_{0}/R=2.85$ are given in table 1. We have also reported the sums of $C_{v}$ of the 8th and 9th dispersion surfaces, below the band-gap, and of the 10th and 11th dispersion surfaces, above the band-gap. We note that the combined valley Chern numbers at Γ, in both $T_{1}$ and $T_{2}$, are zero. Conversely, at K the combined $C_{v}$ for the two dispersion surfaces below the band-gap is −0.5, while for the two dispersion surfaces above the band-gap, it is equal to 0.5. The valley Chern numbers at $K^{\prime }$ are flipped in sign with respect to those at K, so that the Chern number over the whole Brillouin zone is zero, as expected. The non-trivial values of Chern numbers predict the presence of topologically protected valley edge modes, as also demonstrated in the following sections. This is in accordance with what has been observed in other elastic systems (see, for instance, [15,16,28]).

**Table 1.** Valley Chern number $C_{v}$, computed for $a_{0}/R=2.85$.

area	point	disp. surf. 8	disp. surf. 9	**8 + 9**	disp. surf. 10	disp. surf. 11	**10 + 11**
$T_{1}$	Γ	0.5	−0.5	**0**	−0.5	0.5	**0**
	K	−0.5	0	−**0**.**5**	0	0.5	**0**.**5**
$T_{2}$	Γ	0.5	−0.5	**0**	0	0	**0**
	$K^{\prime }$	0.5	0	**0**.**5**	0	−0.5	−**0**.**5**

Concerning the results for the lattice parameter $a_{0}/R=3.25$, given in table 2, we note that the combined valley Chern numbers are zero at all points. This confirms that for $a_{0}/R>3$ edge modes are not topologically protected (*trivial* regime).

**Table 2.** Valley Chern number $C_{v}$, computed for $a_{0}/R=3.25$.

area	point	disp. surf. 8	disp. surf. 9	8 + 9	disp. surf. 10	disp. surf. 11	10 + 11
$T_{1}$	Γ	−0.5	0.5	**0**	0.5	−0.5	**0**
	K	−0.5	0.5	**0**	0.5	−0.5	**0**
$T_{2}$	Γ	0.5	−0.5	**0**	0.2	−0.2	**0**
	$K^{\prime }$	0.5	−0.5	**0**	−0.5	0.5	**0**

## 5. Localized modes in infinite systems

**Figure 6.**
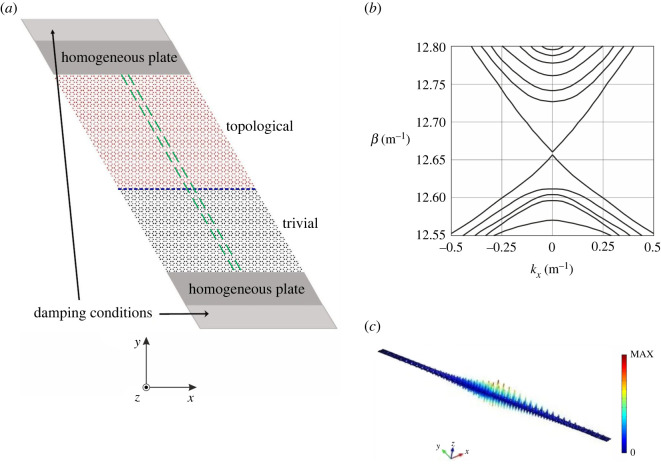
(*a*) Schematic diagram for an infinite plate in the x-direction, comprising a topological ($a_{0}/R=2.85$) and a trivial ($a_{0}/R=3.25$) region of perturbed honeycomb arrays of pins. Damping conditions are introduced near the top and bottom boundaries. The parallelogram macrocell is indicated by dashed lines. (*b*) Dispersion curves for the system in (*a*). (*c*) Example of an eigenmode localized at the interface between the topological and trivial regions, obtained for $\beta =12.69\;{\text{m}}^{-1}$. (Online version in colour.)

Now, we investigate the dispersive properties of a system containing two sub-domains with different perturbations of the pins’ locations: $a_{0}/R=2.85$ (topological region) and $a_{0}/R=3.25$ (trivial region). The number of rows of topological hexagonal cells is 19, while the trivial section contains 14 rows. We assume that the plate is infinite in the x-direction (figure 6*a*), hence quasi-periodicity Bloch–Floquet conditions can be imposed at the boundaries of the parallelogram macrocell, shown by dashed lines. A homogeneous plate (namely, a plate without rigid pins) and a slab incorporating viscous dampers are inserted in proximity of each edge of the system.

Referring to the results reported in §3b, we note that for this choice of the lattice parameters the topological and trivial regions share a common stop-band for $\beta \in [12.68,12.74]\;({\text{m}}^{-1})$. This ensures that localized modes at the interface between the two regions (indicated by a dotted line in figure 6*a*) can be supported.

By performing an eigenfrequency analysis with *Comsol Multiphysics*, which provides the eigenfrequencies for varying wavenumber, we obtain the dispersion curves plotted in figure 6*b*, where we show β versus $k_{x}$ in the vicinity of the unperturbed honeycomb structure’s Dirac point at Γ and $\beta =12.56\;{\text{m}}^{-1}$. We observe that additional modes appear in the mutual band-gap of the topological and trivial regions, and these are located at the interface where the topological and trivial parts meet. An example of an interfacial mode that decays exponentially into the bulk of the system is illustrated in figure 6*c*.

## 6. Interfacial waves in finite clusters

In this section, we demonstrate the ability of these perturbed systems to transport energy with little leakage by modelling plates with finite clusters of pins that act as waveguides. By considering sufficiently large clusters, we can replicate the key properties of the infinite system illustrated in figure 6*a*, and therefore show examples of localized interfacial modes.

**Figure 7.**
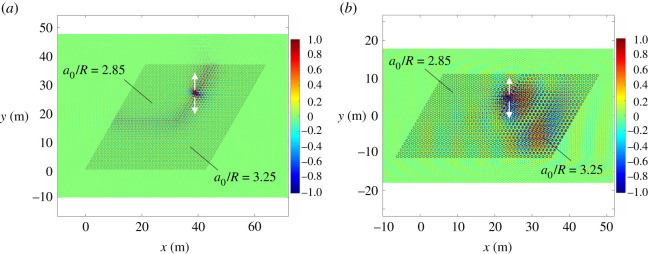
(*a*) Interfacial wave in an elastic plate with a 43×43 cluster of hexagonal cells of rigid pins, with the topological section consisting of 24×24 rows in the top left corner. The wave is generated by a harmonic force (shown by a double arrow) with a frequency parameter $\beta =12.69\;{\text{m}}^{-1}$. The lattice parameters are $a_{0}/R=2.85$ in the topological region and $a_{0}/R=3.25$ in the trivial region. (*b*) For $\beta =12.63\;{\text{m}}^{-1}$, there is no localized wave at the interface, the energy leaks into the trivial part. In this case, the plate incorporates a 42×26 cluster of pins, with the topological section being made of 21×13 rows in the top left corner. (Online version in colour.)

We consider the finite system in figure 7*a*, comprising 43×43 hexagonal cells of rigid pins, divided into a topological ($a_{0}/R=2.85$) and a trivial ($a_{0}/R=3.25$) region. The topological section consists of 24×24 hexagonal cells and is positioned in the top left corner. We apply a point source on the lateral interface (shown by a double arrow in figure 7*a*), characterized by a frequency f=79.814 Hz (corresponding to $\beta =12.69\;{\text{m}}^{-1}$), which is inside the mutual stop-band of the topological and trivial regions. By plotting the displacement field in figure 7*a*, we observe a wave that is localized at the interface, with little leakage into the surrounding medium. The robustness of the system is evidenced by the fact that wave propagation is not affected by the presence of geometrical defects, represented in this case by a corner in the interface.

In figure 7*b*, we show the displacement field when the frequency parameter of the point source is $\beta =12.63\;{\text{m}}^{-1}$, which is in the stop-band for the topological array, but in the pass-band for the trivial one. We observe transmission into the latter section of the system, which highlights that the operating range for the interfacial modes is restricted to the overlapping band-gap for the constituent parts of the system.

In the electronic supplementary material accompanying this paper, we show additional examples of interfacial waves, obtained for a couple of different values of the lattice parameters characterizing the topological and trivial regions of the array of pins.

## 7. Conclusion

We have shown how to perturb honeycomb arrays of pins in elastic plates to obtain either a topological or a trivial band-gap, in analogy with what has been observed in dielectric media containing hexagonal arrangements of cylinders with a different dielectric constant.

First, the study of band inversion and Berry curvatures, localized at specific points in the reciprocal space, has demonstrated that when the ratio of the lattice spacing to the distance between pins in the honeycomb topology is smaller (larger) than 3, a topological (trivial) band-gap opens up in the neighbourhood of the Dirac point of the unperturbed system’s dispersion diagram. In addition, by analysing the dispersion properties of a strip of finite height and periodic in the perpendicular direction, that is also subdivided into a topological and a trivial section, we have observed modes localized at the interface between the two sections for frequencies falling inside the common band-gap of the topological and trivial sections. Finally, we have derived the response of an elastic plate with a large but finite array of pins, again arranged in two sub-regions with topological and trivial band-gaps, to a time-harmonic excitation imposed at the interface between the two regions, and we have observed a localized interfacial wave if the frequency of the excitation lies within the common band-gap. Hence, the proposed model is capable of supporting topologically protected edge and interfacial modes that are immune to backscattering.

We envisage that the results of the present work may have important implications in engineering applications related to wave guiding, vibration isolation and energy harvesting.

## Data Availability

The paper contains no experimental data. The illustrative computations were performed using Wolfram Mathematica (v. 10), Matlab (v. R2021a) and COMSOL Multiphysics (v. 5.5). All results are directly reproducible. Additional examples are provided in electronic supplementary material [59].
